# Supplementary material for: Causal Relationship between Aging and Anorexia Nervosa: A White-Matter-Microstructure-Mediated Mendelian Randomization Analysis
Source: Biomedicines. 2024 Aug 16;12(8):1874. doi: 10.3390/biomedicines12081874 (PMC11351342; doi:10.3390/biomedicines12081874)
Supplement: Supplementary file 1 [file biomedicines-12-01874-s001.zip › Supplementary Figure/Figure S8/Leaveoneout plot of MR analysis for TL to ubm-b-1820.pdf]

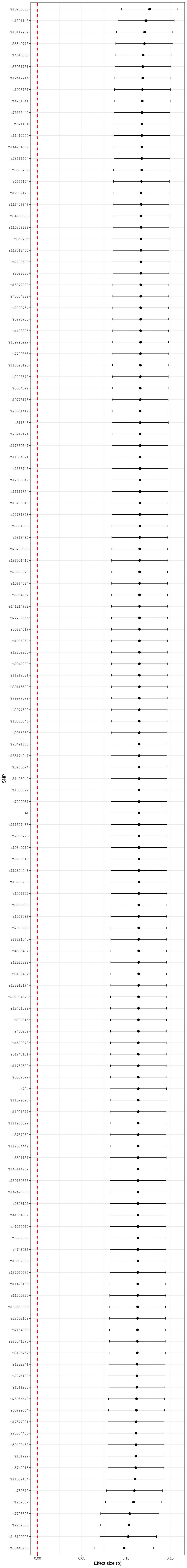
rs10768683
rs1291143
rs10112752
rs35640778
rs4616688
rs56061761
rs12412214
rs1023767
rs4731541
rs76666449
rs871134
rs11412296
rs144204502
rs28577594
rs6536702
rs2555104
rs12932179
rs117407747
rs34550383
rs116863223
rs869785
rs117512405
rs2230590
rs3093888
rs16978028
rs45604339
rs2282764
rs6776756
rs4498805
rs139795227
rs7790856
rs113525195
rs2293579
rs6584579
rs10773176
rs73581419
rs611646
rs76219171
rs117630647
rs11584821
rs2538745
rs17803849
rs11117354
rs13230646
rs66731853
rs6881568
rs9878436
rs73730598
rs137901416
rs28363070
rs10774624
rs6054257
rs141214782
rs77732866
rs80324517
rs1985369
rs12369950
rs9940099
rs11212631
rs80116508
rs79977579
rs2977608
rs10805346
rs9955360
rs78491606
rs185174247
rs3785074
rs61405042
rs1003322
rs7209057
All
rs111527438
rs2056726
rs10840270
rs9600019
rs112394943
rs10905255
rs1907702
rs6669563
rs1957937
rs7099229
rs77231040
rs4695407
rs12925933
rs8102497
rs188918174
rs202034370
rs12451892
rs939916
rs450962
rs4530278
rs61748181
rs11769630
rs6587577
rs4724
rs11579626
rs11991877
rs111950327
rs3767952
rs117034449
rs3891167
rs145114957
rs150150565
rs142426306
rs9398196
rs41304832
rs41269079
rs6659669
rs4743037
rs13062095
rs182059586
rs11426156
rs11699829
rs139669835
rs28502153
rs7164950
rs376641875
rs8105767
rs1332941
rs2276182
rs1611236
rs76065543
rs56799554
rs17677991
rs75664430
rs59409453
rs131797
rs5742915
rs11557154
rs762679
rs932002
rs7705526
rs2967355
rs143190905
rs35446936
SNP
0.00
0.05
0.10
0.15
Effect size (b)
